# Upconverting Nanoparticle Thermometry beyond the Diffraction Limit

**DOI:** 10.1021/acs.accounts.5c00915

**Published:** 2026-03-12

**Authors:** Benjamin Harrington, Ziyang Ye, Laura Signor, Andrea D. Pickel

**Affiliations:** ∇ Materials Science Program, University of Rochester, Rochester, New York 14627, United States; ‡ Department of Chemistry, National University of Singapore, Singapore 117543, Singapore; § The Institute of Optics, 6927 University of Rochester, Rochester, New York 14627, United States; ∥ Department of Mechanical Engineering, University of Rochester, Rochester, New York 14627, United States; ⊥ Walker Department of Mechanical Engineering, The University of Texas at Austin, Austin, Texas 78712, United States

## Abstract

The growing demand for nanoscale temperature measurement capabilities is motivated by diverse applications such as thermal management of microelectronics and batteries, design of plasmonic systems, mechanistic studies of catalysis, and unraveling intracellular processes. Upconverting nanoparticles (UCNPs) are lanthanide-doped inorganic probes that are popular luminescent thermometers, with advantages including well-understood temperature-dependent behavior, broadly tunable excitation and emission wavelengths, and exceptional thermal and chemical stability. Like other optical thermometry techniques, luminescence thermometry provides the desirable capability of remotely collecting the temperature-dependent signal from the far field. Conventional implementations of luminescence thermometry also share a major limitation of other optical thermometry techniques, namely, their diffraction limited spatial resolution. However, in contrast with other optical thermometry techniques, luminescence thermometry also creates an opportunity to leverage certain unique strategies for circumventing the diffraction limit.

In this Account, we discuss our contributions to initiating or building on three major strategies for achieving UCNP thermometry beyond the diffraction limit. Some of these concepts originate from or have direct parallels in the realm of biological imaging, where optical imaging with spatial resolution below the diffraction limit has been a longstanding goal; conversely, others have no direct bioimaging analogy. Exciting an isolated single UCNP with a diffraction limited laser beam enables thermometry with subdiffraction limited spatial resolution governed by the UCNP size, although this approach is inherently restricted to measurements at a single spatial point. We begin by describing our efforts to extend single-UCNP measurements to smaller UCNP sizes and understand how their temperature-dependent emission can be influenced by external factors such as the excitation laser intensity or the surrounding optical environment, the latter of which is exemplified by an investigation of how single-UCNP emission is altered when the UCNPs are placed on various metallic substrates. Next, we show how the principles underlying single-UCNP thermometry can be expanded to sample multiple temperature points within a subdiffraction region by combining different UCNP compositions with spectrally orthogonal temperature-dependent luminescence. As a practical demonstration, we resolve a nearly 20 K temperature difference over a sub-110 nm distance originating from the steep temperature gradient near a laser-heated Ag nanodisk. Finally, we discuss our adaptation of UCNP-based stimulated emission depletion (STED) super-resolution imaging for super-resolution nanothermometry, combining temperature-dependent STED spectroscopy, self-assembled UCNP monolayer formation, and a detection scheme that enables practical scan times. STED nanothermometry can reveal a temperature gradient on a Joule-heated microstructure that is undetectable with analogous diffraction limited measurements, showcasing the power of this approach. We conclude with our perspective on the outlook for UCNP thermometry methods that circumvent the diffraction limit, highlighting both current research needs to further improve the measurement capabilities and strategies that could facilitate broader adoption of these emerging techniques.

## Graphical abstract

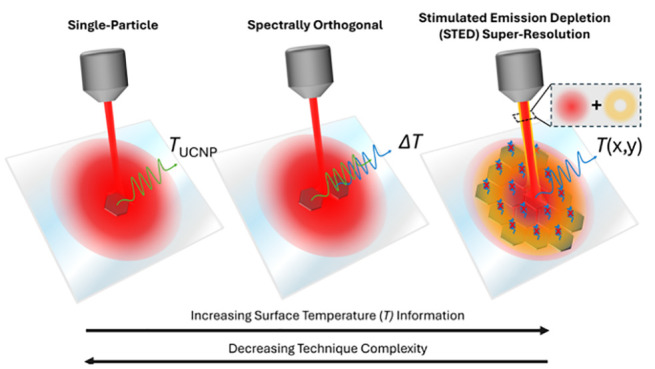

## Key References

- Ye, Z.; Signor, L.; Cohan, M.; Pickel, A.D. Metal surface effects on single upconverting nanoparticle luminescence and thermometry signals. *J. Mater. Chem. C* **2025**, *13*, 116–124.^1^ *The emission intensity of individual UCNPs placed on metal coatings is determined by an interplay between the quenching behavior and reflectance of the underlying metal coating. Conversely, the temperature dependence of the emission is unaltered by the coating material.*
- Harrington, B.; Xiao, Q.; Lin, J.; Johnson, A.; Pickel, A.D. Sampling Sub-Diffraction Temperature Gradients with Spectrally Orthogonal Nanoparticle Luminescence. *ACS Photonics* **2025**, *12*, 11, 6468–6475.^2^ *Tandem pairs consisting of two different UCNP compositions with spectrally orthogonal temperature-dependent luminescence enable sampling of temperature gradients at multiple points within a subdiffraction region. UCNPs separated by ∼ 108 nm can distinguish a ∼ 19 K temperature difference.*
- Ye, Z.; Harrington, B.; Pickel, A.D. Optical super-resolution nanothermometry via stimulated emission depletion imaging of upconverting nanoparticles. *Sci. Adv*. **2024**, *10*, eado6268.^3^ *This study demonstrated a super-resolution nanothermometry technique with sub-120 nm spatial resolution based on highly doped UCNPs that enable STED imaging. STED nanothermometry measurements uncover a temperature gradient on a Joule-heated microstructure that diffraction limited thermometry cannot detect.*

## 1 Introduction

UCNPs offer excellent photostability,^4^ biocompatibility,^5–7^ operating temperatures ranging from cryogenic^8^ to ∼ 1000 K,^9^ and well-established temperature-dependent emission signatures.^10–12^ These and other desirable features have all made UCNPs appealing for thermometry applications. “Ratiometric” thermometry is a common approach typically based on the Boltzmann-distributed relative emission intensity stemming from two closely spaced, thermally coupled energy levels, although emission from nonthermally coupled levels has also been employed.^13^ As the temperature increases, Boltzmann statistics dictate that the higher energy level is populated with greater probability, which manifests as temperature dependence of the UCNP emission spectra. The temperature-dependent ratio is frequently calculated as the integrated emission intensity in the wavelength band corresponding to the transition from the higher energy excited state to the ground state divided by the integrated intensity in the band corresponding to the transition originating from the lower energy excited state. Another common UCNP thermometry metric is the luminescence lifetime,^14^ which generally decreases at elevated temperatures due to the increased probability of phonon-assisted, nonradiative decay.

Among the many UCNP compositions that have been explored for thermometry, NaYF_4_:Yb^3+^,Er^3+^ is by far the most popular.^15^ Here, the Yb^3+^ sensitizer ions absorb 975–980 nm excitation light, while the Er^3+^ activator ions emit temperature-dependent luminescence predominantly at green wavelengths. The temperature-dependent ratio of the integrated emission intensities from the ^2^H_11/2_ to ^4^I_15/2_ and ^4^S_3/2_ to ^4^I_15/2_ Er^3+^ transitions was first established in bulk glasses beginning in the 1980s.^16–18^ The same principle was later extended to lanthanide-doped crystalline nanoparticles,^19^ including biocompatible nanoparticles.^20^ Subsequent studies of UCNP compositions involving various crystalline host matrices and lanthanide dopant combinations have continued to spur new thermometry methods, including several approaches discussed later in this Account. Today, most implementations of UCNP thermometry remain diffraction limited – which can nonetheless provide submicron spatial resolution, sufficiently high for many advanced use cases – but a growing number of frontier applications demand even higher spatial resolution.

Over the last several decades, the development of nanothermometry techniques has been motivated by trends including the ongoing miniaturization of electronic devices,^21,22^ the development of new memory technologies,^23,24^ the proliferation of plasmonics,^25^ and increased investigation of biological processes at the micro and nanoscale.^26–29^ Scanning thermal microscopy techniques that use modified atomic force microscope (AFM) tips as nanoscale temperature probes have been the primary mechanism for achieving nanoscale temperature mapping to date,^30–32^ along with a modest number of near-field optical microscopy approaches.^33,34^ However, these techniques require operating in physical contact with or extremely close to the sample surface, which can pose challenges for accurately determining the sample temperature. Meanwhile, optical thermometry techniques including thermal emission,^35^ thermoreflectance,^36,37^ Raman,^38^ and traditional luminescence-based techniques^39^ allow remote probing and collection of the temperature-dependent signal from the far field but are fundamentally constrained by diffraction limited spatial resolution. Combining the desirable qualities of far-field optical thermal metrology with nanoscale spatial resolution could facilitate the development of improved thermal management solutions, finer control over biological and catalytic processes, and deeper fundamental understanding of nanoscale thermal transport phenomena.

UCNP thermometry is strongly intertwined with biological applications due to the biocompatibility of UCNPs and their extensive development as bioimaging probes.^5–7^ There are also direct ties between bioimaging and the concept of UCNP thermometry with spatial resolution below the diffraction limit, given that optical imaging with subdiffraction limited spatial resolution has been a longstanding bioimaging goal. This goal has been realized through optical super-resolution imaging techniques,^40^ including stochastic single-molecule localization methods^41^ and deterministic approaches like STED,^42^ which were collectively recognized with the 2014 Nobel Prize in Chemistry. Historically, these super-resolution techniques have relied on probes like organic dyes and fluorescent proteins, which have multiple characteristics that impede their translation to thermometry. Specifically, these probes generally lack robust temperature-dependent emission signatures, are often restricted to limited operating temperature ranges, and require extremely high laser intensities to selectively deplete their emission. Beginning with the 2017 demonstrations by Liu et al.^43^ and Zhan et al.^44^ that highly Tm^3+^-doped UCNPs enable STED imaging at far lower laser intensities, the rapid growth of UCNP-based super-resolution bioimaging approaches over approximately the past decade has created many new possibilities for adapting super-resolution imaging for nanothermometry.

The nanothermometry methods our group has developed draw substantial inspiration from super-resolution bioimaging. Along many axes, the requirements of these techniques overlap, while in other regards nanothermometry has distinct needs compared to those of nanoscale bioimaging. Our group seeks to both leverage relevant prior research on UCNP-based super-resolution bioimaging when our goals align with this work, while also carefully considering alternative concepts that are beneficial for nanothermometry but have no obvious parallel in bioimaging. In this Account, we use the terminology “super-resolution” to refer solely to techniques that truly circumvent the diffraction limit – specifically, those with resolution better than the Abbe criterion – and thus enable continuous optical imaging or temperature mapping with subdiffraction limited spatial resolution. We adopt the phrasing “beyond the diffraction limit” to encompass other approaches that in some manner provide temperature information at subdiffraction length scales but, strictly speaking, do not meet this definition of “super-resolution.” The UCNP thermometry capabilities developed by our group also build on one another: our single-UCNP measurements provide a foundation both for simultaneously acquiring emission from multiple isolated UCNPs, as required for multipoint measurements, and detecting single-UCNP-level signals while concurrently adding a second, coaligned laser beam, as required for STED measurements that enable continuous mapping.

## 2 Single-Particle Thermometry

In the context of single-particle luminescence thermometry, there exist two major methods for circumventing the diffraction limit. Attaching a single particle to a probe tip that is scanned over a sample allows for temperature mapping (Figure 1a), while placing an isolated single particle on a sample facilitates single-point temperature measurements (Figure 1b). Using a single particle can enable measurements with spatial resolution below the diffraction limit if only one particle falls within the excitation laser beam spot.

**Figure 1:**
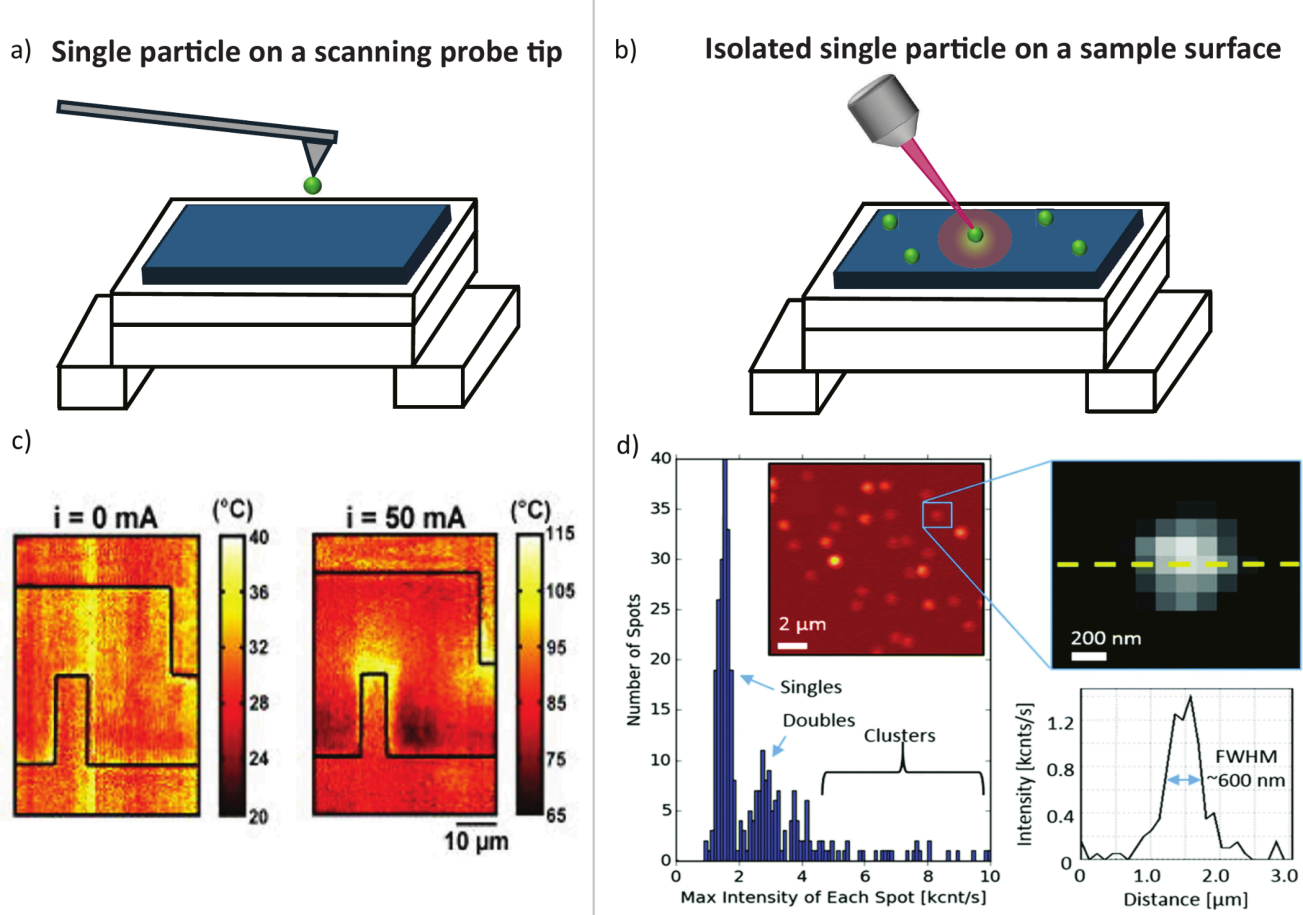
Single-UCNP thermometry with subdiffraction limited spatial resolution can be achieved by (a) attaching a UCNP to a scanning probe tip for temperature mapping or (b) isolating UCNPs on a sample surface for single-point measurements. (c) Temperature maps obtained using the technique depicted in (a) with no current and ∼50 mA of current passing through the circuit. Reprinted from ref^45^ with the permission of AIP Publishing. (d) Histogram of emission intensities used to optically identify single UCNPs, along with an APD image and intensity linecut for a single UCNP. Reproduced from ref^51^ with permission from the Royal Society of Chemistry.

While a large proportion of UCNP thermometry work is focused on biological applications, interestingly, the earliest demonstration of thermometry via a single upconverting particle applied a scanning thermal imaging approach to a semiconductor integrated circuit. Aiguoy et al. attached a single Yb^3+^/Er^3+^ codoped fluoride glass particle approximately a micron in size to the tip of an AFM probe and obtained temperature maps of a microelectronic circuit with and without an applied electrical current.^45^ Hot spots approximately 30 °C higher than the coldest portions of the structure were visible near the corners when a current was applied (Figure 1c). Saïdi et al. later expanded on this method by using a single 200 nm Yb^3+^/Er^3+^ codoped PbF_2_ UCNP attached to a tungsten tip to explore how variations in the thickness of a SiO_2_ layer between a Si substrate and a Ti Joule heater with nanoscale constrictions altered the surface temperature profile, demonstrating that thinner oxide layers produced localized hot spots at the heater constrictions.^46^ While such scanning thermal imaging approaches enable temperature mapping with subdiffraction limited spatial resolution, unknown thermal resistances and parasitic heat sinking via the probe can create challenges for accurately quantifying the sample temperature. Because UCNP thermometry fundamentally records the UCNP temperature, ensuring that this temperature accurately reflects the sample temperature is critical. Our prior work has shown that applying conformal coatings to modify the thermal contact between individual UCNPs and underlying substrates has no effect on the measured temperature.^47^ Similarly, temperatures we measured using isolated single UCNPs, self-assembled layers, and randomly dispersed aggregates show strong agreement,^3^ further confirming that the UCNPs are in good thermal contact with the underlying sample.

In some cases, full temperature maps are unnecessary and single-point measurements are sufficient. In these situations, UCNPs can be deposited sparsely on the surface to ensure individual UCNPs are spaced sufficiently far apart such that their diffraction limited emission spots will not overlap. The resulting spatial resolution of the temperature measurement is determined solely by the diameter of the UCNP. Spin coating dilute solutions of UCNPs results in a random dispersion of particles; however, probing the temperature at specific positions may require precise placement. If a UCNP does not naturally land in the region of interest, deterministic positioning techniques such as nanomanipulation using an AFM tip can be employed,^48,49^ although these methods are often low-throughput and challenging to implement.

Optically identifying single UCNPs is nontrivial since emission spot size is a poor indicator of whether the spot originates from a single UCNP or multiple UCNPs. To address this challenge, we employ a statistical approach previously demonstrated by other researchers based on the emission spot brightness.^50,51^ For example, Kilbane et al. used an avalanche photodiode (APD) to image hundreds of isolated UCNP emission spots and analyzed a histogram of their luminescence intensities^51^ (Figure 1d). The first peak of the histogram provides a characteristic single-UCNP intensity, enabling in situ optical identification.

Zimmers et al. were the first to utilize a single upconverting particle positioned on a sample surface for thermometry, applying this approach to investigate the insulator–metal transition of VO_2_.^52^ Earlier work showed that this transition could be triggered by a voltage or current without an observable global temperature rise. However, temperature measurements performed with a ∼ 1 μm Yb^3+^/Er^3+^ codoped fluoride glass particle positioned within a microscale VO_2_ channel revealed that localized heating occurs, indicating that Joule heating plays a critical role in driving the transition.

Subsequent single-UCNP thermometry research focused on employing smaller UCNPs to further enhance the spatial resolution of the measurements, building on advances in the realm of single-UCNP imaging. Improved synthesis strategies and the development of optimized compositions informed by advanced modeling helped facilitate imaging of individual UCNPs as small as sub-10 nm.^4,50,53^ Despite fruitful efforts to enhance single-UCNP brightness, decreasing the UCNP diameter inevitably requires high excitation intensities, typically ∼ 10^4^–10^6^ W cm^–2^ for single UCNPs tens of nm in diameter. Kilbane et al. investigated the temperature-dependent ratio *r*(*T*) originating from the well-studied green Er^3+^ emission of individual 20 × 20 × 40 nm^3^ NaYF_4_:Yb^3+^,Er^3+^ UCNPs (Figure 2a).^51^ Even at high excitation intensities, *r*(*T*) for these UCNPs still followed the typical Boltzmann-type behavior observed at lower intensities. Kilbane et al. further demonstrated that these sub-50 nm UCNPs exhibit excellent particle-to-particle uniformity in *r*(*T*), enabling the development of a batch calibration curve that can then be applied to other, uncalibrated UCNPs from the same batch, rather than calibrating each UCNP individually.

**Figure 2:**
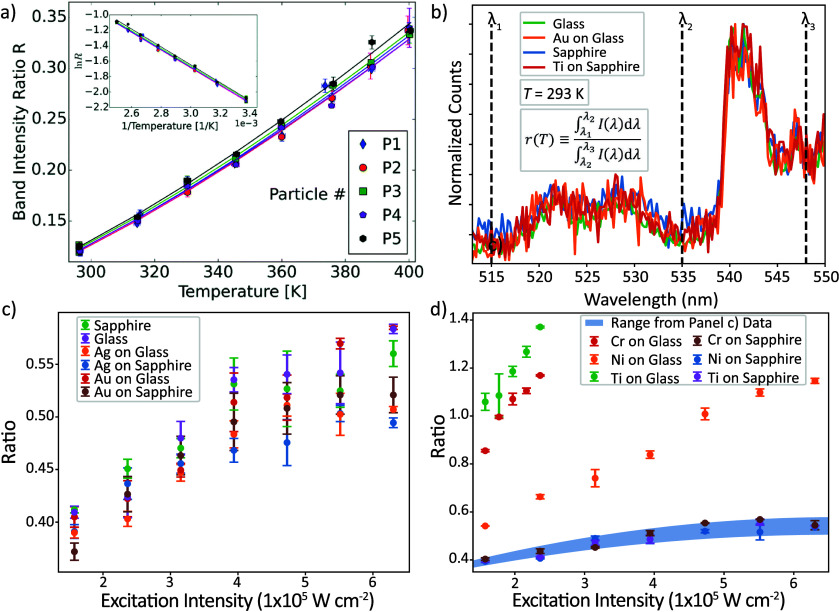
(a) *r*(*T*) for five individual UCNPs, showing excellent particle-to-particle uniformity. Reproduced from ref^51^ with permission from the Royal Society of Chemistry. (b) Normalized room temperature spectra for single UCNPs on different underlying substrates and metal coatings are in good agreement. (c) Changes in *r* with excitation intensity for single UCNPs on glass and sapphire substrates, with and without metal coatings. Despite the wide-ranging substrate thermal properties, all data sets agree well, indicating a nonthermal effect. (d) The blue shaded region represents the nonthermal effect from (c). An additional increase in *r* attributed to laser heating is observed only for the more absorbing coatings on the lower thermal conductivity substrate. Panels (b)-(d) are reproduced from ref^1^ with permission from the Royal Society of Chemistry.

However, with increasing excitation intensity, Pickel et al. observed that the ratiometric signal *r* (detailed in the introduction) from individual 50 × 50 × 50 nm^3^ NaYF_4_:Yb^3+^,Er^3+^ UCNPs also increases, implying a temperature rise of more than 50 K.^47^ This value is over an order of magnitude higher than even a highly conservative thermal model predicts. To investigate this seeming contradiction, Pickel et al. varied the thermal environment surrounding the UCNP, yet in all cases the “self-heating” effect persists. Thus, they concluded that the phenomenon is not a result of poor heat dissipation but is instead a nonthermal artifact caused by increased radiative and nonradiative relaxation from higher-energy excited states, an interpretation supported by rate equation modeling. Consequently, ratiometric thermometry measurements should be carried out using the lowest excitation intensity that still yields an adequate signal-to-noise ratio.

Later work considered not only how nonthermal excitation intensity effects can distort UCNP thermometry signals, but also how the surrounding environment can affect the temperature-dependent emission. Van Swieten et al. demonstrated that when UCNP thermometers are placed in inhomogeneous photonic environments, interference of the direct and reflected UCNP emission can lead to erroneous temperature measurements if such photonic effects are not carefully accounted for.^54^ By placing NaYF_4_:Yb^3+^,Er^3+^ and NaYF_4_:Ho^3+^ UCNPs at controlled distances from an Au mirror using a ramped alumina spacer, Vonk et al. further showed that the same effect could lead to temperature read-out errors as large as 250 K.^55^

These previous studies motivated our group to investigate how the emission intensity and *r*(*T*) of individual NaYF_4_:Yb^3+^,Er^3+^ UCNPs ∼ 27 nm in diameter might be altered by underlying metallic coatings.^1^ We found that the average single-UCNP emission intensity was influenced by the metal film reflectance, but reflectance was not the sole determining factor. For example, Au and Ag have very similar reflectivities, yet single UCNPs placed on Au and Ag films displayed different average emission intensities. This result can be explained by stronger nonradiative decay for UCNPs placed on an Ag film relative to those placed on an Au film, which is evident from the measured lifetime decay curves. We also investigated how the emission spectra change with different underlying metal coatings (Figure 2b) and determined that *r*(*T*) is unaffected. We recorded a nonthermal increase in *r* with increasing excitation intensity similar to that reported by Pickel at al., which we showed is uniform across different metal coatings (Figure 2c). We observed additional increases in *r* for absorbing metal films deposited on the low thermal conductivity substrate, glass, a result attributed to true laser heating (Figure 2d). The largest temperature rise observed is for the Ti-coated sample due to its higher optical absorptance relative to Cr and Ni. These findings highlight the robustness of ratiometric UCNP thermometry on metal surfaces, which is especially relevant for many nonbiological applications, along with various factors that must be considered to achieve accurate single-UCNP temperature measurements.

## 3 Thermometry via Spectrally Orthogonal Luminescence

As detailed in the prior section, sufficiently isolated individual UCNPs can access subdiffraction temperature information at single points. However, the indistinguishable emission from multiple UCNPs of the same composition located within one diffraction limited excitation spot fundamentally prohibits measurement of temperature gradients below the diffraction limit. We recently applied the spectrally orthogonal emission from tandem pairs consisting of two UCNPs of different compositions to probe subdiffraction temperature gradients.^2^ This work represents the first example of utilizing the spectral domain to differentiate the emission from closely spaced luminescent thermometers positioned over a temperature gradient. Existing studies have explored different axes along which the emission from closely spaced probes or emitters can be separated, primarily for the purpose of improving the sensitivity of ensemble UCNP thermometry. This goal is distinct from our application of spectral orthogonality to achieve UCNP thermometry with subdiffraction limited spatial resolution. Nonetheless, aspects of these prior studies are relevant in discussing our use of spectral orthogonality to distinguish the emission from multiple UCNP thermometers.

Beyond spectral differences, Qiu et al. temporally differentiated signals from upconverting PbS quantum dots (QDs) and NaYbF_4_:Tm^3+^@NaYF_4_:Yb^3+^@NaYF_4_:Nd^3+^ UCNPs, two thermometry probes both with emission around 810 nm^56^ (Figure 3a). Taking advantage of their different luminescence lifetimes (∼ns for QDs vs ∼ μs for UCNPs), a robust ratiometric thermometer was developed and applied for intratumoral thermometry.

**Figure 3:**
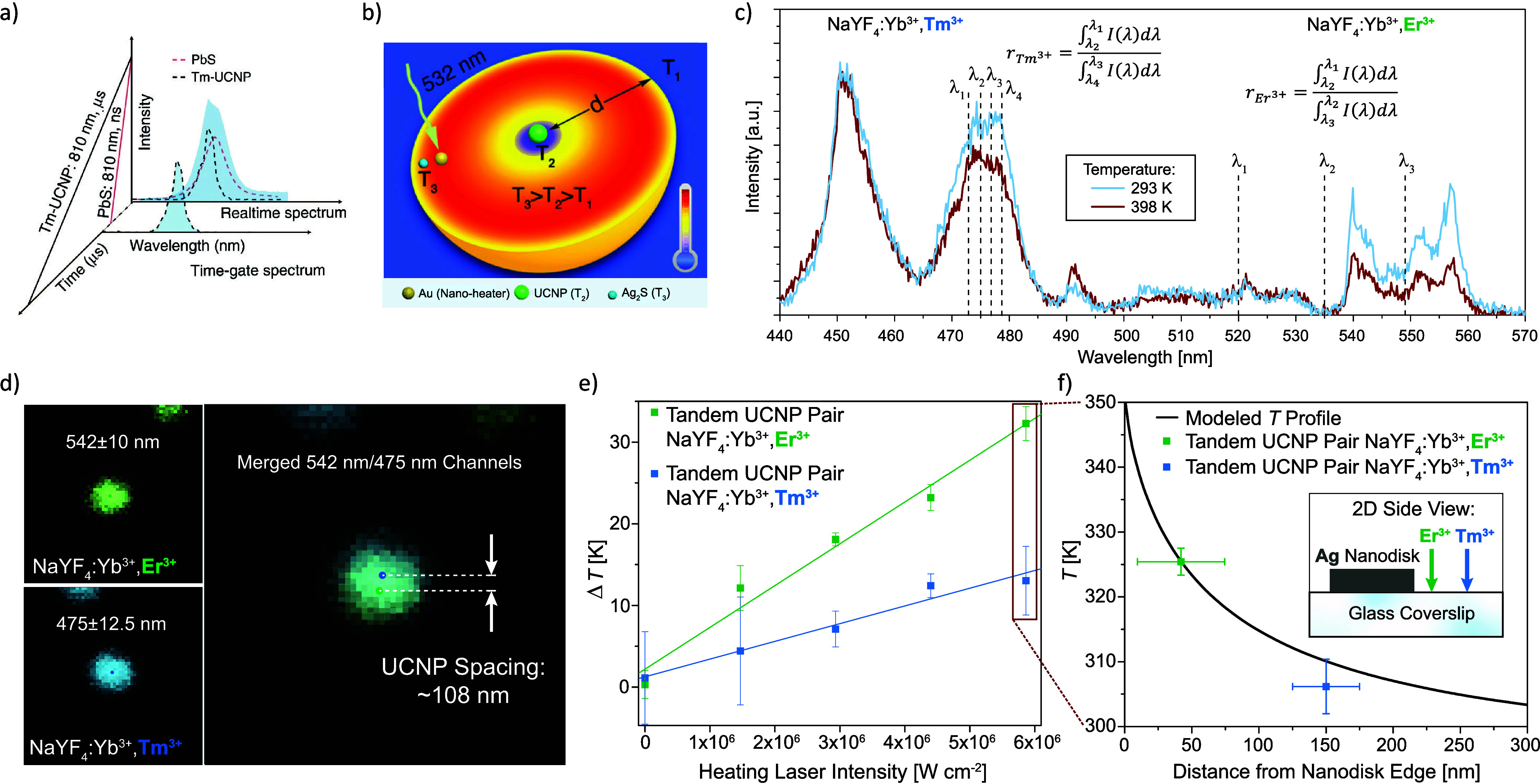
(a) Two upconversion thermometry signals separated in the time domain. Reprinted with permission under a Creative Commons CC BY 4.0 license from ref.^56^ Copyright 2020 The Authors. (b) Schematic of nanocomposites containing UCNPs and QDs that produce spectrally orthogonal thermometry signals. Reprinted with permission from ref.^60^ Copyright 2020 American Chemical Society. (c) Temperature-dependent emission spectra from a tandem UCNP pair. (d) APD images of the emitted luminescence from a tandem UCNP pair on a glass substrate near the edge of an Ag nanodisk. The tandem pair consists of one NaYF_4_:Yb^3+^,Er^3+^ and one NaYF_4_:Yb^3+^,Tm^3+^ UCNP spaced ∼108 nm apart, as determined using the centroids of the UCNP emission spots. (e) Temperature rises measured by the tandem UCNP pair from (d) as a function of the heating laser intensity. (f) Finite element model of the temperature profile together with the highest measured temperature values from (e). Panels (c)-(f) are reprinted with permission under a Creative Commons CC BY 4.0 license from ref.^2^ Copyright 2025 The Authors.

Dual-color thermometers often involve core–shell UCNP structures with layers containing different trivalent lanthanide (Ln^3+^) species. Previous studies have combined spectrally orthogonal, nonthermally coupled emission from different Ln^3+^ dopants to create a single ratiometric thermometry signal with improved relative sensitivity *S*_r_. UCNPs consisting of a NaYF_4_:Yb^3+^,Er^3+^ core with NaYbF_4_:Tm^3+^ and NaYF_4_:Nd^3+^ shells were evaluated for ratiometric thermometry based on the ^1^D_2_ to ^3^F_4_ Tm^3+^ transition (blue luminescence) and the ^4^F_9/2_ to ^4^I_15/2_ Er^3+^ transition (red luminescence).^57^ A ∼ 1.4x improvement in the maximum *S*_r_ was achieved compared to the typical sensitivity for NaYF_4_:Yb^3+^,Er^3+^ UCNPs. Brites et al. combined the emission from separate Yb^3+^/Er^3+^ (green luminescence) and Yb^3+^/Tm^3+^ (blue luminescence) codoped NaYF_4_ and NaGdF_4_ UCNPs distributed within a polymer film to increase *S*_r_.^58^ Like our work, this approach relies on a single excitation source for two different UCNP compositions. Our group also separately leveraged spectral orthogonality to simultaneously perform ratiometric UCNP thermometry and monitor a chemical reaction via Raman spectroscopy, but this method again provides only a single spatial temperature measurement.^59^

To access subdiffraction temperature information, each Ln^3+^ dopant must instead produce an independent ratiometric thermometry signal and be isolated to distinct regions on a sample or within a nanocomposite. To realize the latter case, Lin et al. formed a nanocomposite comprised of an inner core of NaYbF_4_:Er^3+^@NaYF_4_ UCNP thermometers surrounded by Au nanoparticles embedded in mesoporous silica and an outer layer of Ag_2_S QD thermometers^60^ (Figure 3b). The Au nanoparticles were optically heated and the luminescent thermometers were used to measure the temperature at the center and edge of the nanocomposite. Here, only temperature gradients within the nanocomposite are accessible, and such probes thus cannot measure external temperature gradients on a sample surface.

In our recent work, we formed tandem UCNP pairs consisting of individual NaYF_4_:Yb^3+^,Er^3+^ and NaYF_4_:Yb^3+^,Tm^3+^ UCNPs.^2^ Placing the UCNPs along a steep temperature gradient and recording the spectrally orthogonal, temperature-dependent Er^3+^ and Tm^3+^ emission excited by a single laser beam lets us access subdiffraction temperature information at multiple discrete locations, an intermediate regime between single-particle and super-resolution measurements. While recording information at only a few discrete points is largely irrelevant in the context of bioimaging, obtaining even two temperature data points can in some cases yield far more useful information than a single-point measurement, such as measuring the temperature drop across an interface or gaining insight into where heat is generated when this information is not known a priori. The UCNP diameter ultimately determines how close together the temperature probes can be placed. Additionally, the size of the UCNPs relative to steepness of the thermal gradient should be considered, since spatial averaging across the UCNP diameter can influence the recorded temperature. Placing the UCNPs too far apart can cause the emission intensity to drop off sharply due to the Gaussian excitation laser profile, while identifying ratiometric thermometry signals located at nearby, but nonoverlapping, wavelengths is important to reduce chromatic aberrations that can introduce nonthermal artifacts in *r*(*T*).

We applied our technique to study the nanoscale temperature gradient near the edge of a laser-heated Ag nanodisk on a glass substrate. We located a region with a NaYF_4_:Yb^3+^,Er^3+^ UCNP ∼ 42 nm from the edge of the nanodisk and a NaYF_4_:Yb^3+^,Tm^3+^ UCNP ∼ 150 nm from the edge. We used the common ratiometric thermometry signal based on green Er^3+^ emission and a ratiometric thermometry signal for Tm^3+^ involving transitions between the Stark sublevels of the ^1^G_4_ and ^3^H_6_ manifolds that result in blue luminescence, the latter of which had not previously been validated at the single-UCNP level (Figure 3c). Notably, the emission spots from the tandem pair almost entirely overlapped spatially, but by incorporating appropriate bandpass filters in front of our APD, we easily distinguished the two UCNP signals (Figure 3d). We determined the positions of the UCNPs relative to the nanodisk by overlaying the UCNP emission centroids located from APD scans and a widefield image of the nanodisk.^2^ We then measured a ∼ 19 K temperature difference across the ∼ 108 nm distance between the two UCNPs under the maximum heating laser intensity applied (Figure 3e and 3f). Like single-UCNP thermometry, our spectral orthogonality approach would benefit from improved UCNP positioning capabilities. However, unlike single-UCNP placement schemes where all UCNPs should be sufficiently isolated (>1 μm apart), here we require that UCNPs of the same composition are isolated from one another but UCNPs of different compositions are located within <0.5 μm, creating additional complexities. Our technique can be deployed to sample temperature gradients that lack the axial symmetry of that generated by the laser-heated Ag nanodisk, although doing so would naturally impose more stringent UCNP positioning requirements.

## 4 Super-Resolution Nanothermometry

Although the UCNP thermometry techniques described in the prior sections can probe temperature with spatial resolution below the diffraction limit, they largely yield data only at one or two discrete locations, making temperature mapping impractical. Our STED nanothermometry technique provides a fundamentally different pathway for optical temperature mapping at the nanoscale. This technique originated from STED imaging, which was developed and refined for subdiffraction bioimaging.^61^ Much of the instrumentation for STED imaging is identical to that for confocal microscopy, but unlike confocal methods, a second, doughnut-shaped depletion laser beam at a different wavelength is used to drive excited emitters back to the ground state via stimulated emission. Consequently, only the central region emits spontaneously, effectively constricting the point spread function and decoupling spatial resolution from the excitation beam size.

Despite its promise, STED imaging faces limitations, including high depletion laser intensities that can cause photobleaching and unwanted heating. A notable breakthrough came in 2017, when it was demonstrated that cross-relaxation in heavily Tm^3+^-doped UCNPs can greatly enhance depletion efficiency,^43,44^ opening a new avenue for developing STED nanothermometry by combining the ratiometric thermometry capabilities of UCNPs with lower-intensity depletion. Intense Tm^3+^-Tm^3+^ cross-relaxation redistributes the excited state populations and establishes population inversion, enabling stimulated emission under 808 nm illumination that impedes the upconversion process. However, establishing a robust STED thermometry platform requires design considerations distinct from those in bioimaging. We identified key challenges and provided proof of concept using heavily Tm^3+^-doped UCNPs (NaYF_4_:20% Yb^3+^,8–10% Tm^3+^) as an efficient STED probe newly applied to thermometry.^3^

The first challenge arises from practical signal collection considerations. Traditional STED imaging employs oil immersion objective lenses with numerical apertures (NAs) exceeding 1.4. Although ideal for bioimaging, such objectives are less suitable for thermal studies because oil contact and the short working distance (typically <0.2 mm) can perturb the local temperature field by introducing heat sinking, undermining the far field signal collection advantage of optical thermometry. Employing a dry air objective mitigates these effects but inevitably reduces the NA and signal collection (Figure 4a). To compensate, we deliberately increased the UCNP size to ∼ 134 nm to boost the luminescence intensity, particularly for the weaker spectral bands that are both suitable for thermometry and have high depletion efficiencies. While the reduction in NA slightly increases the single-UCNP STED imaging emission spot size, the spatial resolution of temperature mapping is determined by the depletion efficiency rather than the UCNP size. Moreover, unlike conventional STED imaging, which requires only one strong, depletable emission channel, robust optical thermometry, especially ratiometric approaches, demands two emission bands that simultaneously exhibit a thermally sensitive intensity ratio and can both be efficiently depleted. Based on these criteria, we identified the ∼ 492 nm emission peak, which has been observed for other Tm^3+^-doped UCNPs^44,62,63^ but not yet definitively attributed to a particular Tm^3+^ transition, and the ∼ 514 nm emission peak, attributed to the ^1^D_2_ to ^3^H_5_ Tm^3+^ transition, as suitable candidates for ratiometric STED nanothermometry (Figure 4b and 4c). The integrated intensity ratio of the ∼ 492 nm peak versus the ∼ 514 nm peak displays an increase with temperature that is well-approximated as linear over the ∼ 293 to 400 K range considered. We validated the depletion efficiency and super-resolution imaging capabilities of these two spectral features at the single-UCNP level (Figure 4d), demonstrating consistent *r*(*T*) read-outs in both STED and confocal modalities from room temperature up to 400 K.

**Figure 4:**
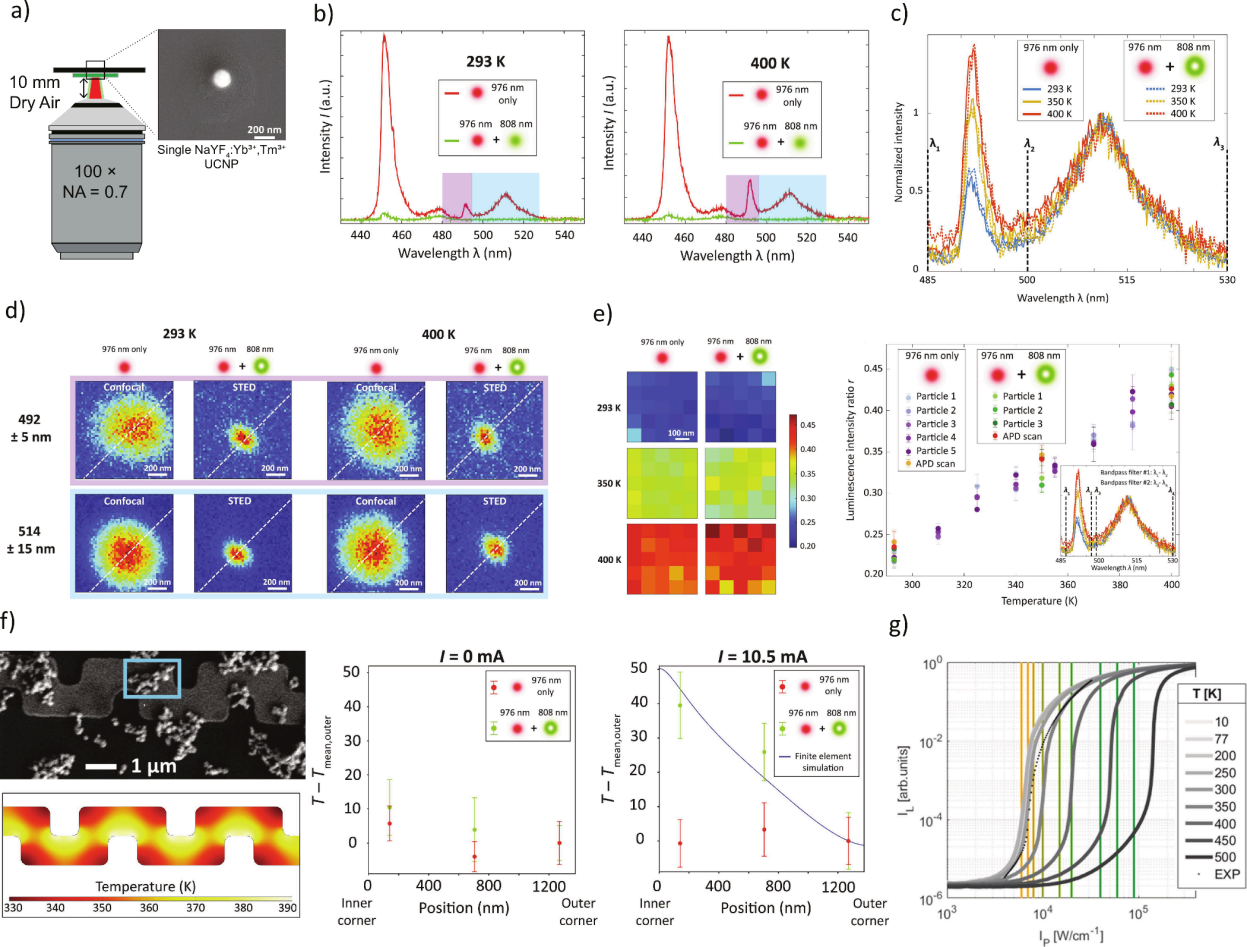
(a) Super-resolution measurements using a custom-built STED microscopy and spectroscopy system, including a dry air objective lens with a 10 mm working distance. (b) Single-UCNP emission spectra at 293 and 400 K when only a 976 nm Gaussian laser beam was applied and when both 976 and 808 nm Gaussian laser beams were applied. (c) Temperature-dependent normalized single-UCNP spectra acquired in diffraction-limited and STED modalities. (d) Confocal and STED imaging at 293 and 400 K of a single ∼134 nm diameter UCNP using bandpass filters corresponding to the ∼492 nm and ∼514 nm emission peaks. (e) Left: *r*(*T*) maps acquired from a UCNP multilayer using APD scans and two bandpass filters in confocal and STED modalities at different uniform sample temperatures. Right: *r*(*T*) values obtained from APD scans and spectroscopy show excellent agreement. (f) Left: UCNPs drop cast on a NiCr serpentine heater line that generates a temperature gradient from the inner to the outer corner when an electrical current is applied. Right: *T – T*_mean,outer_ measured at three locations with and without an applied current, where *T*_mean,outer_ is the mean temperature from 12 measurements near the outer corner. Only the STED measurements can detect a Joule heating-induced temperature gradient across the heater line. Panels (a)-(f) are adapted with permission under a Creative Commons CC BY-NC 4.0 license from ref.^3^ Copyright 2024 The Authors. (g) Simulated and room temperature experimental data showing the temperature-dependent excitation intensity threshold for the onset of PA. Reproduced from ref^64^ with permission under a Creative Commons CC BY-NC-ND license. Copyright 2021 The Authors.

To translate single-UCNP measurements into practical temperature mapping, we formed self-assembled monolayers and multilayers of UCNPs using an interfacial self-assembly method.^65^ In parallel, we implemented a detection scheme featuring an APD and carefully selected bandpass filters to ensure fast and accurate ratio acquisition. This configuration reproduces *r*(*T*) values closely aligned with the spectroscopic calibration (Figure 4e). Furthermore, our detection scheme reduces the total measurement time from several hours to a few minutes, highlighting the practicality of STED nanothermometry for high-resolution temperature mapping applications. To showcase the technique’s resolving power, we designed a Joule-heated serpentine microstructure via finite element simulation and judicious material selection to produce *a* > 40 K temperature gradient across only ∼ 1 μm between the inner and outer corners of the serpentine. Whereas conventional diffraction-limited thermometry detected no measurable gradient, STED nanothermometry clearly resolved this sharp temperature variation (Figure 4f). This approach achieves spatial resolution better than ∼ 120 nm, with a minimum detectable temperature change of approximately ± 0.8 K at a fixed location and an overall measurement uncertainty of ∼ ± 10 K, arising primarily from particle-to-particle variation and the use of a batch calibration procedure.^3^

Even as these results underscore the promise of STED nanothermometry, key challenges remain. The doughnut-shaped depletion beam intensity, although lower than for traditional probes, remains fairly high and can therefore introduce additional local heating. Moreover, although modest reductions in the excitation intensity can dramatically reduce the required depletion intensity, this favorable nonlinearity competes with the nonlinear nature of upconversion: decreasing the excitation intensity inevitably leads to a steep drop in emission intensity, a trade-off that must be carefully managed. Downconversion materials offer a complementary approach because their emission originates from a single-photon process and thus scales linearly with excitation intensity. Liang et al. showed that NaYF_4_:Nd^3+^ nanoparticles exhibit efficient near-infrared downshifting emission and luminescence depletion at intensities an order of magnitude lower than UCNPs.^66^ The same emission bands have been used for ratiometric thermometry, suggesting an even lower-intensity pathway to STED nanothermometry.

Another potential strategy is to exploit photon avalanching (PA) nanoparticles. These materials exhibit extremely nonlinear emission vs excitation intensity scaling (maximum reported >500 ^67^), enabling nanoscale imaging without an additional depletion beam.^68^ Nanoscale PA imaging technically does not beat the diffraction limit in that the achievable sub-100 nm resolution aligns with the modified Abbe criterion for nonlinear emitters;^69^ nonetheless, we discuss nanoscale PA imaging in this section since the resulting spatial resolution is comparable to that of super-resolution methods. However, the excitation intensity threshold for the onset of PA behavior is strongly temperature-dependent due to the importance of multiphonon relaxation and other phonon-assisted processes^64^ (Figure 4g). The degree of nonlinearity observed in the emission vs excitation intensity behavior can also vary with temperature. This intrinsic temperature sensitivity offers both an opportunity and a challenge: while it could be applied for sensitive nanothermometry, the spatial resolution may also become a complicated function of temperature.

## 5 Conclusion and Outlook

As summarized in this Account, our group has introduced or expanded multiple strategies for realizing UCNP thermometry beyond the diffraction limit. This work spans single-point measurements, multipoint measurements, and continuous mapping capabilities, and there generally exists a trade-off between the amount of spatially resolved temperature information that can be obtained and the instrumentation and measurement complexity. Bioimaging remains a major source of inspiration, yet we also carefully consider where the requirements of nanothermometry diverge from those of bioimaging, both so that we can harness strategies that have no clear use case in bioimaging and understand where nanothermometry imposes additional, stringent measurement demands. Whenever possible, we also aim to produce practical demonstrations of the techniques we develop, providing tangible examples of how nanothermometry can access temperature information that diffraction limited measurements cannot.

Surveying the literature reveals that UCNP thermometry beyond the diffraction limit remains far less common than traditional diffraction limited UCNP thermometry. While the already submicron resolution of diffraction limited UCNP thermometry may be more than sufficient for many applications, numerous other applications could benefit from higher spatial resolution. Undoubtedly, the measurement challenges associated with the techniques we have discussed limit their broader adoption. All involve weak signals, single- and multipoint UCNP thermometry can require precise placement of individual UCNPs, and super-resolution methods can require complex wavefront shaping. These obstacles dictate several future research needs: brighter UCNPs, scalable strategies for placing individual UCNPs, and simpler optical instrumentation for super-resolution or related continuous temperature mapping approaches. Scalable UCNP placement strategies will be particularly critical for multipoint temperature measurements where the UCNPs must be placed at specific locations on a sample with no positioning flexibility. Bright UCNPs will advance all measurements but would be especially beneficial for further reducing the laser intensities required for STED nanothermometry.^3^ Fortunately, these needs are again well-aligned with those of bioimaging or other fields, and much progress has been made to date; indeed, the advent of continuous wave STED^70^ was pivotal to our eventual entry into this line of research. Beyond UCNPs, other nanoparticles that produce temperature-dependent luminescence or Raman signals also facilitate single-particle thermometry.^71^ Our demonstration of super-resolution nanothermometry currently remains singular, while other prospective continuous mapping approaches like PA are unique to UCNPs. These attributes together with UCNPs’ ability to withstand wide-ranging and challenging operating conditions continue to drive their widespread use in nanothermometry applications. We expect to see continued progress in high-resolution UCNP imaging that inspires new directions in UCNP nanothermometry, and we look forward to the ongoing development of UCNP thermometry beyond the diffraction limit and its application to a wide array of scientific and engineering problems.
